# Research Progress on Helmet Liner Materials and Structural Applications

**DOI:** 10.3390/ma17112649

**Published:** 2024-05-30

**Authors:** Xingyu Zhang, Bin Yang, Jinguo Wu, Xin Li, Ronghua Zhou

**Affiliations:** 1 School of Automotive & Rail Transit, Nanjing Institute of Technology, Nanjing 211167, China; 18473256993@163.com (X.Z.); wujg8848@163.com (J.W.); lixin1990@nuaa.edu.cn (X.L.); 2 Zhiwu Yunxiang (Nanjing) Information Technology Co., Ltd., Nanjing 210031, China; 18611073228@163.com

**Keywords:** helmet liner, energy absorption performance, composite materials, bionic structure, additive manufacturing

## Abstract

As an important part of head protection equipment, research on the material and structural application of helmet liners has always been one of the hotspots in the field of helmets. This paper first discusses common helmet liner materials, including traditional polystyrene, polyethylene, polypropylene, etc., as well as newly emerging anisotropic materials, polymer nanocomposites, etc. Secondly, the design concept of the helmet liner structure is discussed, including the use of a multi-layer structure, the addition of geometric irregular bubbles to enhance the energy absorption effect, and the introduction of new manufacturing processes, such as additive manufacturing technology, to realize the preparation of complex structures. Then, the application of biomimetic structures to helmet liner design is analyzed, such as the design of helmet liner structures with more energy absorption properties based on biological tissue structures. On this basis, we propose extending the concept of bionic structural design to the fusion of plant stalks and animal skeletal structures, and combining additive manufacturing technology to significantly reduce energy loss during elastic yield energy absorption, thus developing a reusable helmet that provides a research direction for future helmet liner materials and structural applications.

## 1. Introduction

In contemporary Chinese cities, rapid population growth and urbanization, the continuous expansion of commercial and industrial centers has led to a sharp increase in car ownership, and the problem of traffic congestion has spread from big cities to smaller and medium-sized ones, making urban transportation a hot topic of widespread concern at all levels of society. Therefore, cycling, as an environmentally friendly, economical, and healthy way to relieve traffic congestion, has gradually become the first choice for people to travel. In Kunming, for example, electric bicycles have effectively replaced many urban car trips [1]. According to statistics, the proportion of cycling trips has increased from about 5% a few years ago to more than 10%, continues to grow, and is expected to continue to grow in the future [2]. According to annual data of domestic road traffic accidents from 2019 to 2022 released by the National Bureau of Statistics (Table 1) [3], the total number of bicycle and motorcycle traffic accidents increased from 19.3% in 2019 to 20.2% in 2022. This increasing trend has aroused people’s concerns about bicycle and motorcycle safety, and the traffic safety of cyclists has become an important issue of social concern. Every year, tens of thousands of vehicle collisions worldwide result in the death of a large number of two-wheeler cyclists, while causing huge economic losses [4]. In automobile-two-wheeler crashes, head injuries are the most common type of injury that cause fatalities and serious injuries among cyclists [5,6,7]. Studies have shown that wearing a helmet can effectively reduce head injuries suffered by cyclists in traffic accidents [8,9,10]. In an analysis of the clinical features of 15,345 patients with traumatic brain injury (TBI) across 35 hospitals in Henan Province [11], the proportion of injuries caused by riding electric bicycles was 25.5% (2481/9731), which has become one of the main causes of traffic accidents. In addition, it has been reported that about 55% of cycling deaths are due to head injuries [12]. For cyclists, helmets are their only protection against injuries such as TBI [13,14,15,16]. Jiangsu Province was the first to formally implement the “Regulations on the Administration of Electric Bicycles in Jiangsu Province” [17]. In the following years, similar laws were also implemented in various provinces and cities [18,19,20,21]. Studies have shown that mandatory helmet-wearing legislations have been associated with a substantial decrease in the number of e-bike roadway fatalities, with a year-on-year reduction in fatalities of 2.21% [22].

Common life protective helmets are mainly composed of four parts: shell parts, adjusting devices, hard lining parts, and soft lining parts [23]. Hard lining parts and soft lining parts are collectively referred to as liners. In these components, the liner absorbs energy through viscoelastic compression to relieve acceleration to the head [24], thereby reducing the risk of head injury for the rider [25]. The majority of cushions are typically composed of synthetic cellular materials, like expanded polypropylene (EPP) and expanded polystyrene (EPS), and the density of the cushion material is often adjusted to achieve the best energy absorption effect [26]. In recent years, scholars at home and abroad have devoted themselves to studying the head protection and shock absorption performance of various advanced materials and innovative structures implanted in helmets, starting with the application of helmet liner materials and structures. In current innovation research, researchers are exploring lightweight and heat-dissipating helmets design concepts. For example, they are experimenting with the use of materials such as multi-foam, plant fiber, and innovative fabrics. On the other hand, in the field of bionic structures, researchers have designed many structures that can significantly improve protective effectiveness. They have not addressed the issue of increased helmet weight and discomfort. Therefore, light bionic structures, such as thin-walled structures, cell structures, and auxiliary structures, should be introduced into the helmet liner. While there is an increasing emphasis on novel helmet designs, there is also a growing focus on helmet quality. European ECE R22.05 [27] is a safety standard for motorcycle and bicycle helmets that aims to ensure that helmets meet a strict set of requirements during the design and manufacturing process to provide optimal head protection. The standard stipulates that the maximum head acceleration cannot exceed 300 g at any time, or 150 g for a period exceeding 15 ms [28]. However, this widely adopted standard still neglects some important issues, such as the fact that the test standard only considers linear movement of the head and not rotational acceleration. Rotational acceleration is more dominant in destructive intracranial injuries in head injuries. Therefore, it is crucial to consider reducing the acceleration of the rotating head through legislative provisions in all certified helmet testing standards to improve the performance of helmets and better regulate the market.

This article reviews the most current designs of existing helmet liner materials, structures, and biomimetic structures and their protective properties, with a particular focus on the application of bio-inspired structures and materials for energy absorption in protective helmets. The paper will look at the innovativeness, practicability, and feasibility of various design options, and, based on this, will provide an outlook on the possible challenges, limitations, and future directions for the field at the present time.

## 2. The Development of Helmet Liners

**Figure 1:**
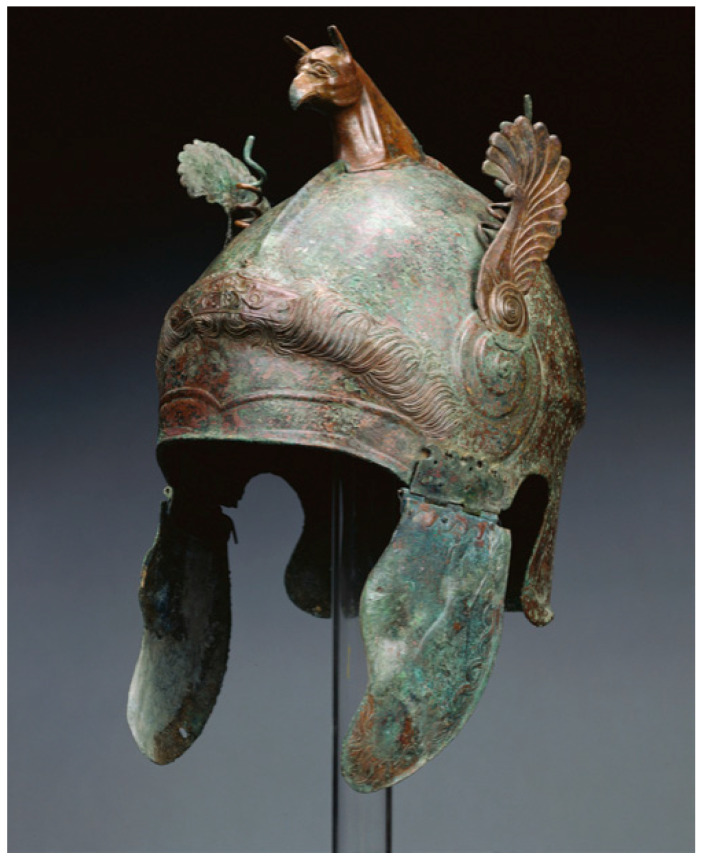
Ancient Greek bronze helmet. Digital image courtesy of the Getty’s Open Content Program. J. Paul Getty Museum. Public domain [30].

**Figure 2:**
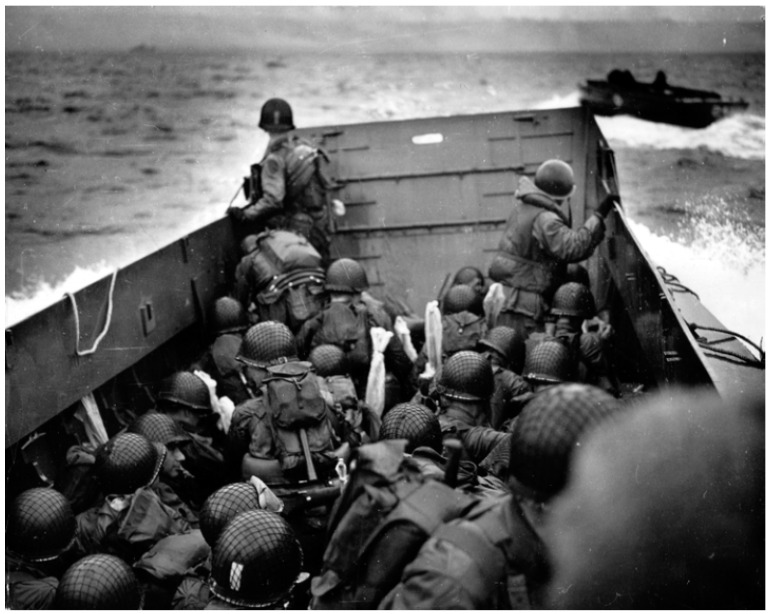
M1 helmet. Photography by Conseil Régional de Basse-Normandie, National Archives USA. Public domain [30].

**Figure 3:**
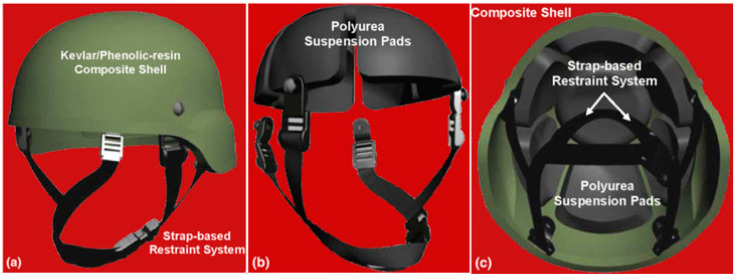
Advanced combat helmets (ACH): (**a**) external side view; (**b**) suspension system side view; and (**c**) suspension system bottom view [31].

Helmets have long been employed as a main type of protection, covering the head from weapons and any kind of penetration [29]. Simultaneously, a helmet liner is a kind of soft liner located inside the helmet and, in ancient times, warriors usually added some soft materials, such as leather, fabric, and grass, as helmet liners inside the helmet. Hard leather, brass, or iron were the most common materials used to make ancient war helmets. The ancient Greeks’ bronze helmets are notable examples (Figure 1). Although these bronze helmets were able to defend against direct attacks, such as swords and arrows, they had to be improved due to the poor energy absorption and weight of the bronze material. With the advent of the Middle Ages, warfare technology advanced, and the helmet design became more complex. The helmet liner has been improved accordingly, with thicker fabrics, liner, or cotton, etc., to provide better protection and reduce pressure on the helmet, as well as to enhance the comfort of wearing the helmet. With the development of metallurgical technology, helmet materials have gradually evolved from metal products to more advanced materials, such as steel and alloys. With the development of metallurgical technology, helmet materials have gradually evolved from metal products to more advanced materials, such as steel and alloys. In the 1940s, military and civilian helmets were commonly built of a robust steel casing with a plastic and cotton fiber interior. During World War II, the US Armed Forces used the M1 steel helmet in both European and Pacific theaters (Figure 2). The gasket material was made of high-elastic rubber material, and the whole helmet was relatively lightweight, allowing the wearer to move more flexibly, reducing the burden on the head and neck, and improving the survivability and mobility of soldiers in combat. In the early 1970s, DuPont succeeded in developing a new synthetic fiber called Kevlar, an aramid polymer that had many beneficial properties, including low density, high strength, toughness, high temperature resistance, and ease of processing and molding. The fiber’s ability to absorb kinetic energy from shrapnel was 1.6 times that of nylon (a condensed polymer) and twice that of steel. In the early 1980s, the U.S. military introduced the Personnel Armor System for Ground Troops (PASGT) helmet, which was composed of up to 19 layers of Kevlar^®^ K29 (DuPont, Wilmington, DE, USA). However, with in the evolution of warfare, operational environments, and the development of new materials, the U.S. Army designed the advanced combat helmet (ACH) using Kevlar^®^ K129 (DuPont, Wilmington, DE, USA) to replace the PASGT helmet. The ACH helmet is composed of an altered edge cutout for reduced protection surfaces, an external composite shell that is 7.8 mm thick and composed of stronger Kevlar^®^ K129 fibers and lower content phenolic resin, and has a unique assembly of distinct foam liners, referred to as the “suspension system”, which is strategically positioned on the inside of the helmet and fastened with hook-and-loop fasteners. The shape of the ACH helmet, together with a list of all its basic parts, is shown in Figure 3. During the war in Afghanistan, the U.S. military found bullets could often penetrate Kevlar-made helmet. Therefore, the U.S. military designed an enhanced combat helmet (ECH) made of ultra-high molecular weight polyethylene (UHMWPE) material to replace the popular ACH helmet at that time. Table 2 summarizes some of the relevant parameters of the U.S. Army combat helmet model.

Helmet liners have evolved from ancient leather, fabric, and other soft materials to modern foam plastics, rubber, and other new materials. While continuously improving the helmet’s energy absorption, comfort, and adaptability, they have also significantly improved the helmet’s protective performance, greatly reducing the risk of fatal head injuries.

## 3. Main Materials of a Helmet Liner

### 3.1. Head Protection Performance

When a pedestrian is engaged in a traffic collision, a shock to the head can produce multiple forms of brain damage, which can be separated into focal and diffuse brain injuries based on their clinical symptoms [37]. A focal brain injury is due to linear acceleration of the head, while diffuse brain injury is mainly related to rotational acceleration. As a result, it is critical to lessen the linear and rotational acceleration that the head experiences.

**Figure 4:**
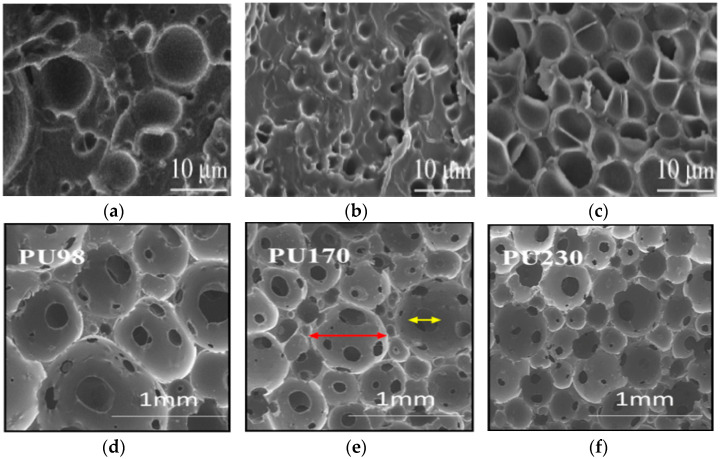
SEM photo comparison of PES foam and PU foam: (**a**) PES at an immersion pressure of 10 MPa; (**b**) PES at an immersion pressure of 12.5 MPa; (**c**) PES at an immersion pressure of 15 MPa; (**d**) PU at a density of 98 kg/m^3^; (**e**) PU at a density of 170 kg/m^3^; and (**f**) PU at a density of 170 kg/m^3^. Cell apertures are shown by yellow arrows, while cell diameters are indicated by red arrows [40,41].

Mosleh et al. [38] suggested an anisotropic EPS composite foam concept to reduce head rotational acceleration, thereby minimizing the risk of brain injury. In comparison to the single-layer EPS foam with a density of 80 kg/m^3^, the 45° tilt impact test results demonstrated a considerable reduction in the peak rotational acceleration and velocity of the EPS composite foam by 44% and 19%, respectively. These impact test results were further analyzed using the overall head injury criterion, which showed that EPS composite foams significantly reduced HIC_15_ (linear head injury criterion with 15 ms impact time), HIC_rot_ (rotational head injury criterion), and HIP_max_ (maximum head impact power) values by 19%, 65%, and 28%, respectively. This suggests that the EPS composite foam idea has significant potential for use in protective helmets. Vanden Bosche et al. [39] used anisotropic polyethersulfone (PES) foam in a bicycle helmet liner and performed oblique impact experiments. The results of this study indicated that the peak rotational and translational acceleration of the PES helmet was almost 40% lower than that of the EPS helmet, suggesting that the PES foam may eventually replace the EPS foam. Figure 4a–c shows the micrographs of the PES foams placed at immersion pressures of 10 MPa, 12.5 MPa, and 15 MPa, respectively [40]. Ramirez et al. [41] manufactured viscoelastic polyurethane (PU) foams with densities of 98 kg/m^3^, 170 kg/m^3^, and 230 kg/m^3^ (as shown in Figure 4d–f), and integrated them into helmet liners for FMVSS motorcycle helmet testing. The results showed that the use of additional PU foam liners on top of existing EPS foam liners successfully reduced peak acceleration by 17%. In the field of equestrian helmets, Cui et al. [42,43] found that the decrease in peak acceleration depends on the contact area, the stress distribution along the cushion thickness, and dissipative plastic energy density (DPED). To lower peak linear acceleration, functional gradient foam (FGF) cushions should be utilized instead of discrete foam layers. To achieve the results of reducing both rotational and linear accelerations, Maheswaran et al. [44] investigated the quasi-static compressive shear response of freestanding vertically oriented carbon nanotubes (VAVNT) foams under different initial precompression conditions. The results demonstrate that VAVNT can tolerate substantial shear strain at a low shear stress level under an enormous compressive shear load. This indicates that VAVNT foam can minimize linear acceleration and rotational acceleration by absorbing normal impact. In motorcycle helmet liner materials, Shuaeib et al. [45] confirmed that EPP foam is widely used due to its multi-impact protection properties and the potential to improve ventilation systems. However, EPP foam is still deficient in head protection due to its material characteristics. In quasi-static compression and shear testing on three VN foams, Bailly et al. [46] discovered that VN foam helmet liners were thought to minimize head rotation acceleration more than EPP foam helmet liners. Chang et al. [47] conducted a study to evaluate the potential protective benefits of copolymer ethylene-vinyl acetate (EVA) foam, a commonly used helmet liner, at various densities against head injury and the dynamic response of a 5.56 mm rifle bullet hitting the cranium when covered by a ballistic helmet. The findings of the study demonstrated that covering the front section of the skull with an EVA liner can significantly reduce acceleration by 600 times. At a specific density of 30 kg/m^3^, EVA foam provided superior protection compared to expanded polypropylene (EPP) foam, reducing peak acceleration at the front of the skull by 36%.

### 3.2. Cushioning Performance

**Figure 5:**
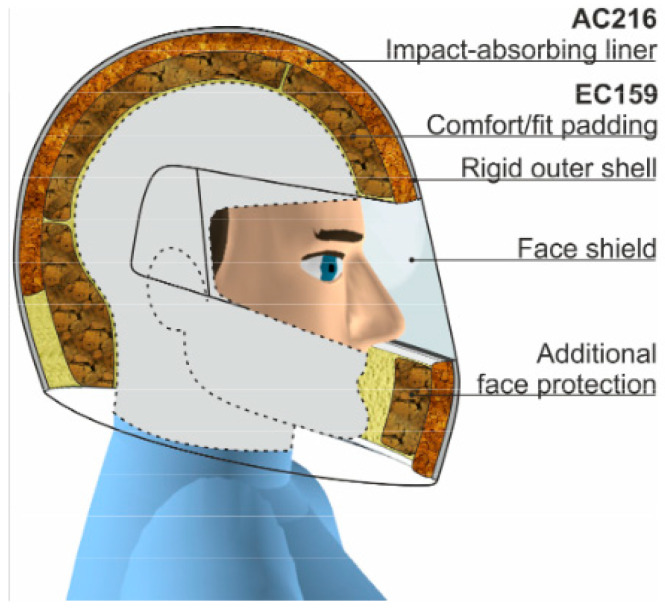
A cross-section of a motor helmet with cork sandwiches applied [54].

The helmet liner is an important component of shock absorption and comfort in the overall helmet design. Wu et al. [48,49] inserted polyethylene (PE) foam liners into both industrial and construction helmets with a series of drop resistance tests. The results showed that the helmets equipped with the PE foam liner were able to withstand a drop impact at a height of 1.52 m, compared to helmets without the liner, which could only sustain a drop impact of 0.61 m. The inclusion of a PE foam liner notably boosted the helmet’s shock-absorbing endurance limit by 145%. Mosleh et al. [38], Kroeker et al. [50], Huang et al. [51], and Drane et al. [52] all confirmed that selecting the right material density or material thickness can significantly improve energy absorption performance. On the other hand, cork is a naturally porous, non-renewable material that is highly resistant to crashes and, due to its viscoelastic behavior, has good resilience after compression, which is an ideal property in multi-impact applications [26]. Buil et al. [53] used cork and its derivatives instead of traditional EPS liners. However, to solve the non-renewable problem of cork materials, Fernandes et al. [26] further investigated whether there were materials that could be synthesized into cork, and finally found that agglomerated cork liners were excellent materials for synthesizing cork liners. Black cork has excellent thermal stability, while agglomerated cork can absorb high energy. Kaczyński et al. [54] discovered that merging two cork materials (agglomerated and black) into a cork composite sandwich structure combines their advantages, but also has good cushioning properties. In addition, Kaczyński et al. [54] applied both agglomerated cork (AC) with a density of 216 kg/m^3^ and expanded cork (EC) with a density of 159 kg/m^3^ in a motorcycle helmet, as illustrated in Figure 5.

### 3.3. Heat Dissipation Performance

**Figure 6:**
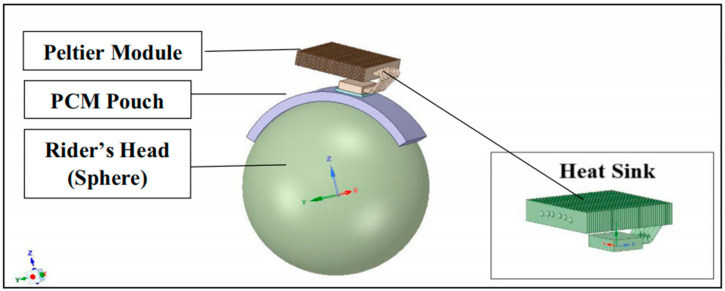
Schematic diagram of a helmet hybrid cooling system application [59].

In recent years, studies have found that the effectiveness of heat dissipation directly affects the comfort of wearing a helmet. In an inventive move to regulate the interior temperature of a motorcycle helmet, Sinnappoo et al. [55] introduced a paraffin phase change material (PCM) cloth to the interlayer between the scalp and the helmet liner. Textile materials have been shown to significantly increase helmet heat dissipation. In particular, it is reported that PCM, when used as textile liner, can significantly reduce the internal temperature of the helmet by 3.8 degrees Celsius. In addition, PCM is capable of absorbing 17.8 W of heat, which is sufficient to mitigate thermal stress within the helmet. Furthermore, after adding carbon nanotube (CNT) nanofillers to a polyurethane (PU) foam, Bhinder et al. [56] observed that the PU foam treated at −5 °C and reinforced with 1.6 wt% CNT oxide absorbed 97% more energy per unit volume under low-speed impact in the drop weight test than EPS. This is attributed to the fact that PU foam’s thermal conductivity is 34% higher than EPS, making it a superior heat dissipator. Kim et al. [57] designed a combination of foam and flocking energy-absorbing material (FEAM) layer elements, which has been shown to combine the advantages of foam materials (lightweight) with FEAM’s excellent impact absorption characteristics, breathability, comfort, perspiration, and heat management. Many studies have started with the heat dissipation properties of materials, but few scholars have studied active cooling methods. Consequently, Jain et al. [58] presented a passive cooling technique based on a multifunctional liner made of eicosane and evenly dispersed graphene oxide nanosheets, as well as a composite thermal liner. The study found that the interaction of the composites allowed 0.3 g of ice oxane to absorb about 2.75 J of heat. The heat absorbed increased proportionally to the thickness of the helmet liner and the concentration of ice oxane. The study also found that the liner, due to its high thermal management qualities, was optimal for the creation of lightweight helmets that effectively dissipate heat. Ebaid et al. [59] created a novel helmet cooling system, amalgamating both PCM and thermoelectric technologies, as shown in Figure 6. The system worked by absorbing excess heat from the user’s head, and then TEC technology was used to cool the PCM, counterbalancing the low temperatures lost during the cooling process. The cooling system maintained a temperature of 19.5 °C when in motion and 25 °C when at rest. The method can be used effectively, and it can be inferred that it enhances the comfort level of the user while wearing the helmet.

## 4. Main Structure of the Helmet Liner

### 4.1. Honeycomb Structure

**Figure 7:**
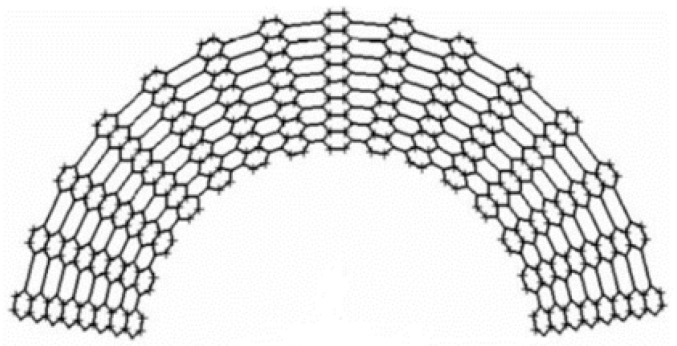
Layered cellular structure [60].

In recent years, research has focused on exploring new helmet liner structures, and honeycomb structures have become a hot research topic due to their stress structures consisting of many hexagonal or hexagonal-like small units. In [60], Kholoosi et al. presented a hierarchical honeycomb structure design (Figure 7). The honeycomb structure was designed as an EPP foam with a thickness of 5.9 mm and covered with ABS (a terpolymer of acrylonitrile, butadiene, and styrene monomers) plastic layer as an energy absorber for the helmet. In contrast to EPS foam, the honeycomb structure exhibits a longer energy absorption period and reduced acceleration of impact force transmission to the user, according to the data. What result would the combination of the two produce? A novel method for fabricating EPS/honeycomb structure foam through an integrated manufacturing process has been proposed by Bhudolia et al. [61]. Three different types of EPS foam samples were subjected to curbstone impact tests: pure EPS foam (PF), hybrid EPS foam with honeycomb in the middle (MHF), and hybrid EPS foam with honeycomb at the top of the foam (THF). The results showed that PF samples absorbed only 82.4% of the impact energy, while THF and MHF samples absorbed 98.5% and 90.8%, respectively. The integration of EPS and honeycomb hybrid liner significantly enhanced energy absorption by up to 20%. The accompanying densification of the foam and the elastic flexural properties of the honeycomb contributed to the absorption of more energy, and the hybrid EPS/honeycomb structure increases energy dissipation and head injury protection. As a result, integrated manufacturing methods have gradually become the main design principle for researchers. Li et al. [62] analyzed the optimal configuration of honeycomb packing geometry and foam density of the helmet liner and conducted drop tests to evaluate its performance compared to a conventional helmet. The study’s findings indicated that the optimized honeycomb liner helmet design significantly reduced peak acceleration, HIC, intracranial pressure, and von Mises by 36.3%, 53.8%, 35.8%, and 46.1%, respectively, when compared to a conventional helmet. In addition to the honeycomb structure, conical structures have also been studied by Teng et al. [63] and Ingrole [64], who discovered new tensile and inflated pillar structures, and spherical structures have been studied by Toboła et al. [65], all of which have good research prospects.

### 4.2. Lattice Structure

**Figure 8:**
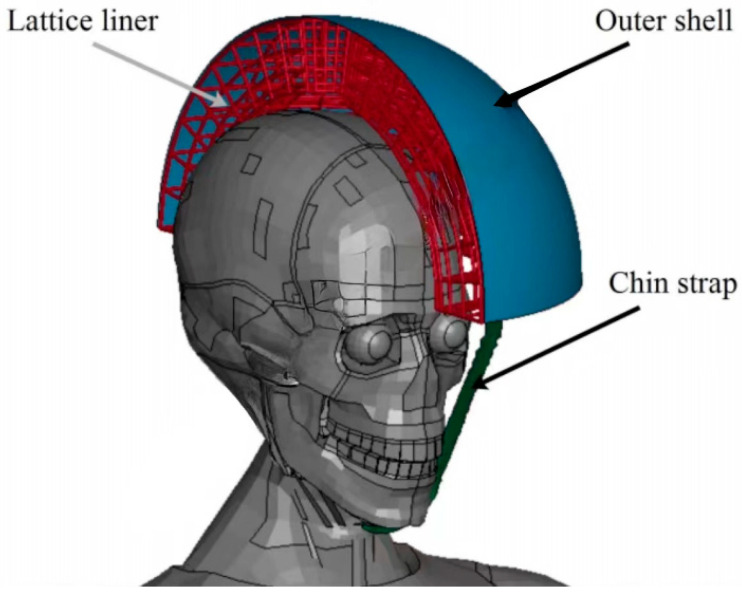
Helmet model with a lattice liner [66].

**Figure 9:**
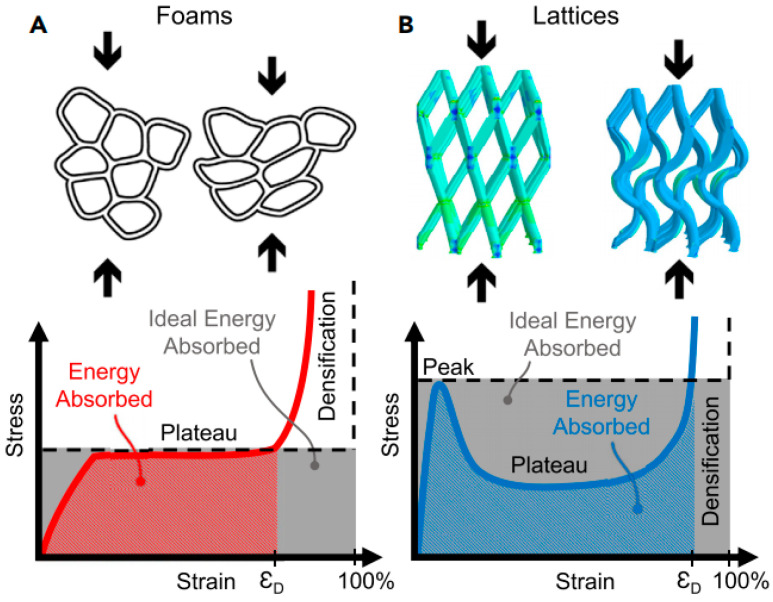
Lattice IA and foam IA compression tests: (**A**) a stress-strain diagram of foam IAs; and (**B**) a stress-strain diagram of lattice IAs [68].

**Figure 10:**
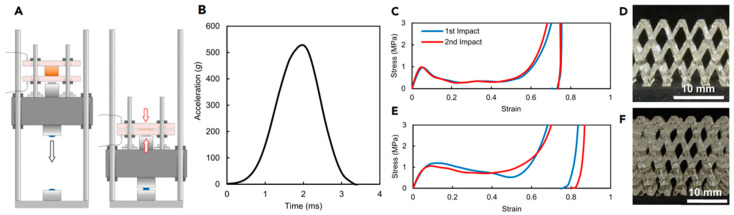
Double anvil dynamic compression test of two lattice IA honeycomb structures with different numbers of unit cells under applied impulsive acceleration conditions mimicking hammer blows: (**A**) double anvil lab bench; (**B**) a pulse acceleration diagram for imitating hammering; (**C**,**E**) stress-strain diagrams for two compressions; and (**D**,**F**) cellular structures of lattice IAs with two different numbers of unit cells [68].

A lattice structure is a common design in helmet liners, which is formed by a series of cross-interwoven lines or grids, similar to the arranged structure of a crystal. The lattice structure can provide better stability and support, and can effectively maintain the shape and structure of the helmet, preventing excessive deformation or damage when exposed to external pressure, thus protecting the wearer’s head. Previously, to verify whether the lattice structure was suitable for helmet liners, Khosroshahi et al. [66] evaluated the performance of the lattice liner topology (Figure 8) as a helmet liner structure. Compared with an EPS liner, the prismatic lattice with a specific relative density of 6% showed superior protection, resulting in the following remarkable reductions: 48%, 37%, 49%, 32%, and 65% in peak linear and rotational acceleration, head injury criteria (HIC), brain strain, and strain rate, respectively. The results showed that the prismatic lattice outperformed the tetrahedral lattice and EPS foam commonly used in helmets in preventing traumatic brain injury (TBI). On this basis, Khosroshahi et al. [67] also studied the feasibility of using layered lattice liners for helmets. The results show that the layered lattice structure reduces the peak linear acceleration of the head by 44% to 66% in the case of direct impact scenarios. In addition, the same lattice liner reduces peak linear acceleration, HIC, and maximum rotational acceleration of the head by 38% to 63%, 31% to 62%, and 55% to 70%, respectively, in the case of oblique impact. Studies have shown that helmets with layered lattice structure liners can be considered a new generation of helmet energy-absorbing liners. On the other hand, as additive manufacturing becomes more efficient, traditionally fabricated structures can be replaced by 3D printing technologies with specific application characteristics. For example, Clough et al. [68] prepared lattice and foam impact attenuators (IAs) using additive manufacturing techniques and evaluated their impact attenuation performance by performing compression experiments on both structures. The bending deformation of the foam IAs cell wall under impact loading is depicted in Figure 9A. The stress-strain response of the foam IAs cell wall is reasonably flat (red solid line), near to the ideal response of a typical flat to a flat impact scenario (black dotted line). IAs always absorb less energy per unit volume than would be required for a flat-to-flat impact; this energy is equivalent to the area under the stress-strain curve. Lattice IAs buckle and exhibit post-peak softening in their stress-strain response, as seen in Figure 9B. When compared to foam, lattice typically has a higher specific modulus, is stronger, and may be made with a greater strain during densification. Furthermore, Clough et al. [68] built cellular structural unit cells of lattice IAs and performed double anvil dynamic compression experiments on two distinct cell counts of lattice IA cellular structures using pulsed acceleration settings resembling hammer impacts. The experimental results indicate (as seen in Figure 10) that the lattice IA honeycomb construction may accomplish a broad variety of dynamic compression behaviors by altering the arrangement density of the unit cell.

### 4.3. Fillable Structure

**Figure 11:**
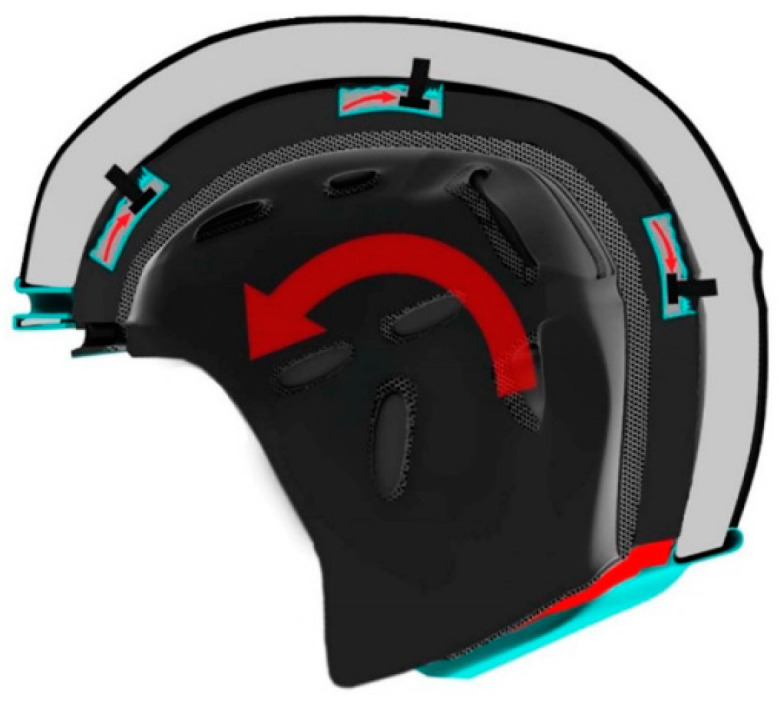
Cross-sectional view of a helmet design with an impact attenuation module [70].

While honeycomb, lattice, and conical structures are frequently raised when discussing helmet liner structures, Goel [69] proposed an innovative design that incorporates a variety of solid or fluid-filled materials, and designed distinct channel structures within the liner. In his research, he explored two different cavity diameters and filled them with water. After many collisions, it was observed that narrower rectangular channels could effectively reduce the peak acceleration by about 25–30%. In addition, glycerin provided the lowest peak acceleration compared to all other materials tested, including glass beads and water. On this basis, La Fauci et al. [70] proposed a shear thickening fluid based on boronated organosilicon to fill the chambers of the liner’s impact energy dissipating liners to form a new type of helmet liner (Figure 11). According to the ECE R22.06 [71], the oblique impact results for the three impact positions were analyzed. The helmet with a liner achieved a 14% reduction in brain injury criteria compared to the helmet without a liner, with values well below standard thresholds. This solution proved to be the most effective at reducing impact energy.

## 5. Bionic Structure

**Figure 12:**
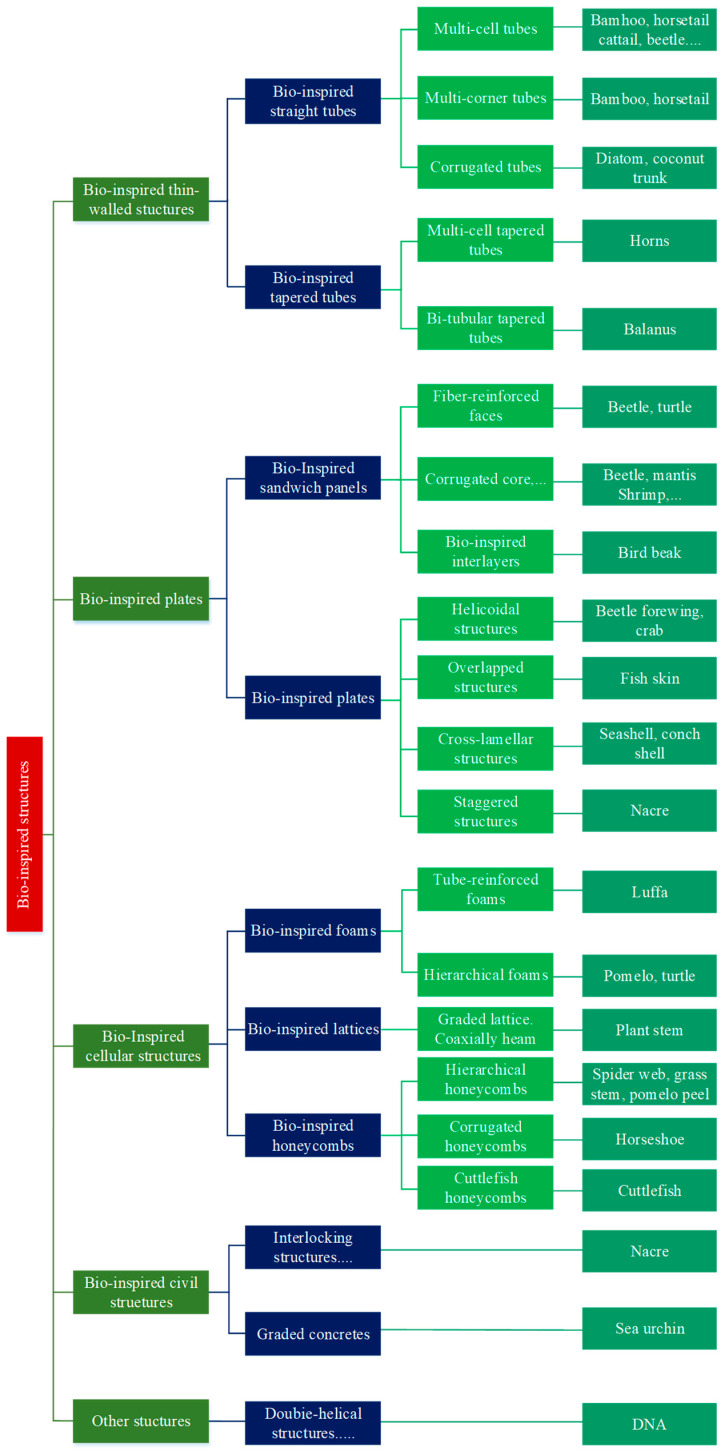
Categories of bio-inspired structures [72].

As a novel technology, biomimetic structures were not widely employed as energy-absorbing materials until about 2000, and the complexity of producing these structures hampered their promotion. Many bionic structures, inspired by biological models and advancements in fabrication processes, have been developed and demonstrated to be successful in energy absorption. Thus, throughout the past several years, bionic structures have been developed more and more rapidly. As seen in Figure 12, San Ha and Lu [72] compiled a wide range of current bioenergy absorption structures and categorized them into several groups according to their form and use. In general, energy absorption in a variety of engineering domains has demonstrated the promise of biomimetic approaches.

**Figure 13:**
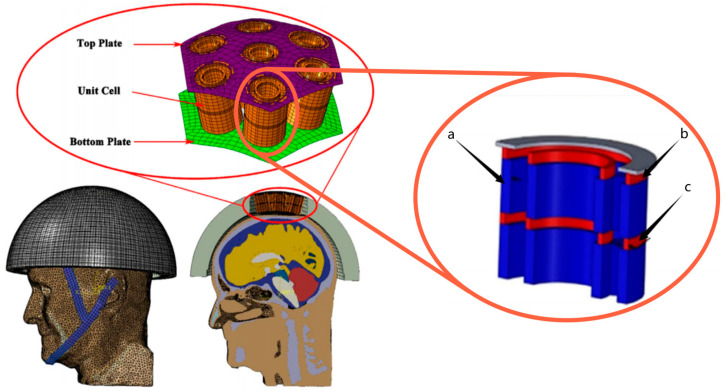
Helmet liner with a multi-layer ponytail biomimetic structure: (**a**) the stem layer; (**b**) the existence of a top hollow layer; and (**c**) the ratio of the hollow layer [79].

**Figure 14:**
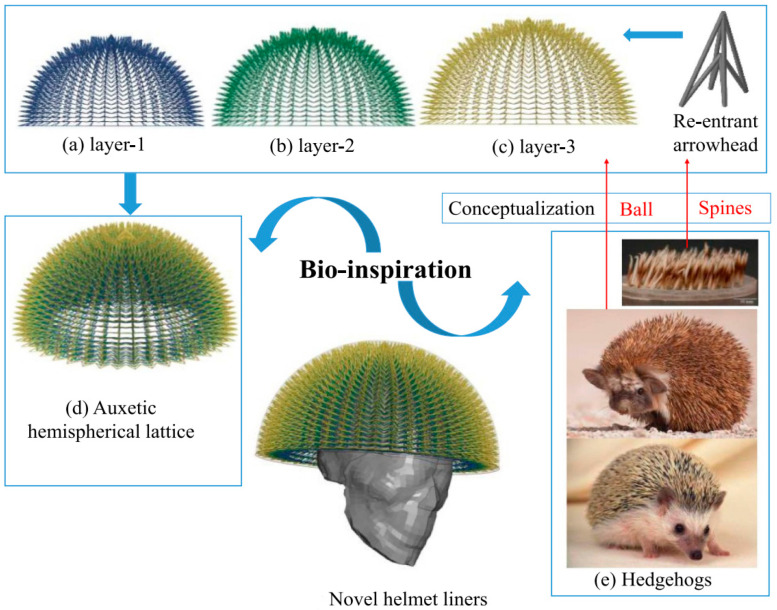
A hedgehog biomimetic helmet [80].

Studies have shown that improving the protective performance of helmets, relevant cushion materials, and structures can be inspired by a wide range of organisms (such as macadamia nuts, grapefruit, and horseshoes) [73,74,75,76]. Motivated by previous studies conducted by Parry et al. [77], Wang et al. [78] discovered that the cattle hoof wall possesses an α-keratin laminar structure. Subsequently, the section of the hoof wall in proximity to the growth line was selected as a suitable test material for nanoindentation and was utilized in the creation of a biomimetic hoof wall via 3D printing. This innovative bionic structure demonstrated its ability to efficiently absorb the energy generated by crack growth and enhance fracture toughness by 55%. This discovery has led to its potential applications in motorcycle helmets and athletic protective gear. In a separate study, Leng et al. [79] proposed a novel multilayered structure based on a multicellular structure mimicking the horsetail stem. Inspired by the elongation and curve of human vertebral multilayers, they created a unicellular horsetail structure with two stem layers and a hollow layer. The stem layer, which resembles spinal vertebrae, efficiently reduces out-of-plane loads, while the hollow layer, resembling a soft intervertebral disc, allows the entire multilayer structure to stretch and bend. The study utilized the presence of the top hollow layer and the thickness of the hollow and stem layers as the main design variables. The experimental results revealed that the inclusion of the top hollow layer reduces the shear stress of the multilayer structure by 30% compared to its absence. Furthermore, increasing the wall thickness of the stem layer from 2 mm to 3.5 mm resulted in an increase in the degree of compressive force at the same value of 16 mm compression by almost 70%, thereby enhancing energy absorption. These research findings indicate that the ideal bionic ponytail structure (shown in Figure 13) can lessen the kinetic energy brought on by rotational and linear accelerations, indicating the structure’s possible use as a helmet liner. This finding also provides a means of exploring new bionic structures created by fusing animal bones and plant stalks. Chen et al. [80] developed a re-entrant arrowhead shape with a negative Poisson’s ratio, designed to mimic the natural spine of hedgehogs, using a strip structure (Figure 14e). For re-entrant arrows, rods with a circular cross-section were used to ensure a stronger resemblance to hedgehog spines. Finally, the re-entrant arrow structure was incorporated as the basic unit cell of the liner, which is composed of three layers of re-entrant lattices creating the bionic liner, as illustrated in Figure 14a–d. The results showed that helmets with a bionic liner were superior to helmets without a liner or those with alternative liner designs. The new helmet design resulted in a 72.65% reduction in head injuries compared to the threshold value. The findings of this study broaden the use of tensile materials and direct the creation of helmet liners that provide improved TBI relief.

In reference to the pyramid-shaped thorns on durian shells, Teng et al. [63,81] devised and created a hemispherical, pyramid-shaped helmet liner out of ABS resin. When the top of the helmet was subjected to a free-fall impact on a flat plate, it displayed 13% more energy absorption than helmets filled with EPS foam, with an average energy absorption of 85.1% This was superior to the potential energy absorption of the EPS foam liner at 81%. Yang et al. [82] designed a new, lightweight bionic bi-directional sinusoidal corrugated sandwich construction, by emulating the jaw-foot form and bi-directional corrugated properties of a peacock mantis shrimp. This construction utilized an upper double sinusoidal arrangement. This structure can reduce stress concentration in the uniaxial out-of-plane compression test and has an energy absorption that is 118% higher than that of the conventional triangular ripple sandwich structure. As a result of Teng’s [63,81] and Yang’s [82] studies, Liu and Xu [83] developed gradient lattice, honeycomb, and bi-directionally interlaced rectangular truncated conical liners. This was accomplished by adjusting the geometrical parameters or pyramidal arrangement concerning the pyramidal spines of durian shells and the bi-directional corrugated configurations of the peacock mantis shrimp’s jaw foot. Finite element simulations of impact were carried out on three types of liners with different pyramidal cell structures, and the kinetic behaviors of liners with diverse structures were studied throughout the impact compression process. Of the helmets tested, the bidirectional staggered rectangular optic cone helmet had the highest specific energy absorption, measuring 1.3 times greater than that of the honeycomb helmet and 2.7 times higher than that of the gradient lattice. This also gives us new ideas for the application of helmet liner structures to animal and fruit internal structure combinations. This revealed that unique bionic liner structures can be designed by combining the shell structure with different animal bone structures.

## 6. Conclusions

Head impact injury is a prevalent traumatic injury, primarily caused by linear and rotational accelerations. Amid societal progression, protective gear such as helmets has been extensively utilized in vehicular accidents, sporting activities, and military sectors. As a crucial component of helmet composition, the material and architecture of the helmet liner are intimately linked to traumatic brain injury, and are also a significant determinant influencing its protective efficacy. This review scrutinizes the evolution of helmet liners across distinct epochs, from its inception to the progression of contemporary structures. From the conventional structure dominated by EPS foam, it has evolved into contemporary composite foam, polymeric materials, and flexible cushioning materials. Concurrently, the structural design of helmet liners has also undergone a metamorphosis from a simplistic honeycomb structure to an intricate lattice structure, inflatable structure, and bionic structure. The design principles and advantages of these novel structures are summarized, and their application to helmet liners necessitates further exploration in the subsequent aspects:

(1) Presently, the materials and structures of most novel helmet liners are solely in the theoretical research phase, and the intricacy and elevated production costs have not been considered. Experiments on helmet crash performance are predominantly confined to straightforward simulation experiments and cannot replicate complex crash scenarios in reality. Moreover, current helmet testing standards lack evaluation of the response to head rotation acceleration. Consequently, there is an immediate necessity to develop testing standards for all novel helmets and to disseminate widely accepted rotation acceleration thresholds.
(2) The comfort of the helmet wearer hinges on the overall weight and breathability of the helmet. Therefore, it is imperative to develop lightweight and heat-dissipating helmets, considering the combination of multi-foam (alleviated weight) and plant fibers (superior breathability), innovative fabrics, and other materials, and further investigating the thermal management properties of helmet liners.
(3) Bionic structures have been demonstrated to outperform traditional structures in energy absorption. Therefore, lightweight bionic structures, such as thin-walled structures, cellular structures, and auxiliary structures, should be incorporated to augment the performance of helmet liners. The design concepts of bionic structures can be extrapolated to the study of composite structures of plant stem structures and animal skeletal structures, reinforcing the application of bioinspired structures and materials in impact energy absorption in protective helmets.
(4) Additive manufacturing technology can utilize the cellular structure with reinforcement and adjustment properties to achieve energy absorption without compromising the helmet liner, which not only enhances the protective performance of helmets but also provides the potential for personalized customization. In the future, novel helmet liner materials and structures with reusable and high energy absorption properties can be developed with the aid of this technology.
(5) By establishing a head helmet-coupled finite element model, a finite element analysis of diverse impact application scenarios can be conducted to explore the propagation characteristics and injury evolution mechanisms of craniocerebral tissue stress waves under impact load. Additionally, a craniocerebral tissue injury assessment model can be established to provide an essential theoretical foundation for the design of helmet liner materials and structures.

## Figures and Tables

**Table 1:** Domestic traffic accident statistics per year from 2019 to 2022 [3].

Type	Total Amount of Traffic Accidents in 2019 (*n* = 247,646)	Total Amount of Traffic Accidents in 2020 (*n* = 244,674)	Total Amount of Traffic Accidents in 2021 (*n* = 273,098)	Total Amount of Traffic Accidents in 2022 (*n* = 256,409)
The total number of bicycle and motorcycle traffic accidents	47,918 (19.3%)	48,400 (19.8%)	54,584 (20.0%)	51,833 (20.2%)
Number of car traffic accidents	159,335 (64.3%)	156,901 (64.1%)	171,941 (63.0%)	157,407 (61.4%)
Number of tractor traffic accidents	1865 (0.8%)	1591 (0.7%)	1502 (0.5%)	1136 (0.4%)
Number of pedestrian and passenger traffic accidents	3432 (1.4%)	3480 (1.4%)	4086 (1.5%)	3907 (1.5%)
Number of other traffic accidents	156 (0.06%)	151 (0.06%)	142 (0.05%)	174 (0.07%)

**Table 2:** Summary of common U.S. Army combat helmet attributes.

Helmet	Material (Shell/Fabric)	Material (Liner)	Chin Strap Fixation	Protection Level, NIJ Standard [32]	Year of Official Commissioning	Reference
M1	Hadfield Steel	Cotton fabric-reinforced phenolic	Two-point chin strap	Not available	1941	[33,34]
PASGT	Thermoset resin/Kevlar^®^ K29 composite	Polyethylene foam	Two-point chin strap	III-A	1980	[35]
ACH	Thermoset resin/Kevlar^®^ K129 composite	Polyethylene foam	Four-point chin strap	III-A	2003	[36]
ECH	UHMWPE HB80 composite	Polyethylene foam	Four-point chin strap	III-A	2014	[36]

## Data Availability

Data are contained within the article.
